# Enhanced CHI3L1 promotes macrophage activation in persistent inflammatory events of ulcerative interstitial cystitis

**DOI:** 10.3389/fimmu.2026.1716297

**Published:** 2026-01-29

**Authors:** Wei Zhang, Yize Guo, Jiawen Wang, Xiaodong Liu, Ting Xie, Yao Li

**Affiliations:** 1 Department of Urology, The Affiliated Hospital of Qingdao University, Qingdao, China; 2 Department of Urology, Fujian Provincial Hospital, Fuzhou, China; 3 Department of Urology, The First Affiliated Hospital of Guangzhou Medical University, Guangzhou, China; 4 Department of General Surgery, Xinqiao Hospital, Army Medical University (Third Military Medical University), Chongqing, China; 5 Department of Cardiology, The Affiliated Hospital of Qingdao University, Qingdao, China

**Keywords:** CHI3L1, glycolysis, interstitial cystitis, macrophage polarization, metabolic reprogramming

## Abstract

**Background:**

Interstitial cystitis/bladder pain syndrome (IC/BPS), particularly the Hunner-type subtype (HIC), is a chronic inflammatory bladder disorder characterized by persistent inflammation and macrophage-driven immunometabolic dysregulation. CHI3L1, a secreted glycoprotein implicated in inflammation and tissue remodeling, is significantly upregulated in HIC and correlates with disease severity, but its mechanistic role in macrophage-mediated persistent inflammatory events (PIEs) remains poorly defined.

**Methods:**

This study integrated multi-omics analyses, including bioinformatics of IC/BPS transcriptomic datasets, a cyclophosphamide-induced IC/BPS mouse model for *in vivo* validation, and *in vitro* functional assays involving CHI3L1 overexpression in macrophages. Transcriptomic, metabolomic, and molecular biology techniques were employed to evaluate metabolic shifts, inflammatory pathways, and transcription factor correlations.

**Results:**

CHI3L1 expression was significantly upregulated in HIC patients, especially those with reduced bladder capacity, and correlated with inflammatory markers (IL-6, TNFα). In macrophages, CHI3L1 overexpression drove pro-inflammatory activation via NF-κB and TNF pathways, promoted glycolysis, and suppressed mitochondrial oxidative phosphorylation (OXPHOS) and aspartate metabolism. Critically, CHI3L1 expression strongly correlated with the transcription factor MYC rather than STAT3 under inflammatory conditions, reinforcing M1 polarization.

**Conclusions:**

CHI3L1 exacerbates PIEs in HIC by reprogramming macrophage metabolism toward glycolysis and sustaining inflammation via MYC signaling. These findings establish CHI3L1 as a central regulator of chronic inflammation in HIC and highlight its potential as a therapeutic target for disrupting pathological immune-metabolic cycles.

## Introduction

Interstitial cystitis/Bladder pain syndrome (IC/BPS) is a chronic bladder disease characterized by pain or discomfort in the bladder area, accompanied by lower urinary tract symptoms and the exclusion of infection and other clearly diagnosable diseases (1). The etiology and pathogenesis of IC/BPS are still unclear, lacking diagnostic gold standards and effective treatment methods. As a heterogeneous clinical syndrome, there are many subtypes of IC/BPS patients, with the two main subtypes being classic IC/BPS (Hunner type IC/BPS, referred to as HIC; characterized by Hunner’s lesion) and non-classic IC/BPS (non-Hunner type IC/BPS, referred to as NHIC; characterized by the absence of Hunner’s lesion) (2). Currently, it is believed that HIC and NHIC patients have different pathogenic mechanisms. For example, the bladder mucosal gene expression profile of HIC patients differs significantly from NHIC patients and normal controls, with the former showing significantly upregulated immune and infection-related pathways (3); meanwhile, the urine of NHIC patients differs little from normal controls, but the urine of HIC patients shows significant upregulation of the JAK-STAT pathway compared to normal controls (4). Consistent with these observations, the response to different treatments varies between HIC and NHIC patients. For example, for HIC patients, the efficacy of transurethral electrocoagulation/electrofulguration (5) and/or submucosal injection of triamcinolone acetonide is better (6), and similarly, this subtype responds better to Cyclosporine A treatment (7). Our research group’s preliminary work has confirmed that immunogenic cell death-related features in the bladder tissue of HIC patients are significantly upregulated compared to NHIC and normal controls, with the persistent presence of chronic inflammation in HIC being associated with its more active inflammatory immune microenvironment (8). Therefore, understanding the mechanism of changes in the bladder immune microenvironment of HIC patients is key to solving this clinical problem.

Macrophages, as the regulatory vanguards of the inflammatory immune microenvironment, play an important regulatory role in tissue remodeling and the resolution of inflammatory responses. Studies suggest that the regulatory mode of macrophages in the immune system is related to tissue type and individual development (9). As mediators of tissue homeostasis, macrophages can convert into specific subtypes (primarily M1 or M2 subtypes) and exert specific regulatory functions depending on the different microenvironments they encounter (10, 11). In inflammatory bladder diseases, macrophages play important roles, including bacterial clearance during bladder infection (12). After macrophage activation, its metabolic pathway shifts from oxidative phosphorylation (OXPHOS) to glycolysis (13, 14); this metabolic switch from OXPHOS to aerobic glycolysis can be mediated by M1 stimulation to meet the upregulation of pro-inflammatory cytokine expression and rapid energy supply (15). Our research group’s previous studies have also shown that resident macrophages in bladder tissue are associated with HIC, and activated macrophages may mediate the sustained infiltration of inflammatory cells in the bladder of HIC patients (16).

In order to resolve the above-mentioned confusion, in this study, our research group integrated the research methods of bioinformatics analysis, transcriptomics, metabolomics, and molecular biology to propose new possibilities regarding the mechanisms of the persistent inflammatory events (PIEs) mediated by activated macrophages in HIC patients.

## Materials and methods

### Integration analysis and selection of DEGs in IC/BPS transcriptome dataset

The “SVA” package was used to merge two IC/BPS transcriptome datasets (GSE11783 and GSE57560) to eliminate batch effects. Genes with a logFC > 2 and adjusted p-value < 0.05 were used as the threshold for identifying differentially expressed genes (DEGs). Pathway enrichment analysis of DEGs was conducted using Metascape (http://metascape.org) (17).

### Construction of IC/BPS mouse model

Female C57BL/6J mice (8–12 weeks, N = 16) were raised in a controlled environment with constant temperature, humidity (24 ± 2°C and 50 ± 5%), and a 12-hour light-dark cycle, with food and water available ad libitum. All mice were purchased from Charles River Company (Beijing, China). All experiments were approved by the Animal Experimental Ethics Committee of the Institute of Biophysics of Chinese Academy of Sciences (NO: syxk2021096), and complied with the National Institutes of Health Guide for the Care and Use of Laboratory Animals (National Institutes of Health publication no. 85–23; revised 1985). Mice were randomly divided into experimental and control groups, with 8 mice in each group. A classical IC/BPS mouse model was established by intraperitoneal injection of cyclophosphamide (50 mg/kg; Sigma-Aldrich, USA) and the cyclophosphamide solution was prepared using sterile saline as the solvent. The experimental groups (IC) were intraperitoneally injected with cyclophosphamide on days 1, 4, and 7, while the control groups (NC) received an equivalent amount of sterile saline. After completing behavioral tests on day 14, the mice were deeply anesthetized via inhalation of 5% isoflurane and then euthanized by cervical dislocation, and bladder tissues were harvested and stored at -80°C.

### Pain threshold detection

Bladder pain symptoms of IC/BPS animal models cannot be objectively measured, and currently, mechanical pain testing methods are often used as alternatives (18, 19). We used the most commonly used von Frey filaments (NC12775-99, North Coast, USA) to assess the mechanical withdrawal threshold (MWT) of mice through the Up-down method (20). In simple terms, mice were allowed to acclimate to the testing apparatus for 30 minutes, then a vertical stimulus was applied to the plantar surface of the right hind paw using the filaments, with the filaments forming a “C” or “S” shape maintained for approximately 6–8 seconds considered a successful stimulus. Positive responses such as licking or scratching the stimulated area were marked as “X”, while negative responses were marked as “O”. The stimulus gradients used were 0.008 g, 0.02 g, 0.04 g, 0.07 g, 0.16 g, 0.4 g, 0.6 g, and 1g. If there was no response to six consecutive stimulations of 1g, it was recorded as 1g. Finally, a formula was used to calculate the 50% MWT (20). MWT testing was conducted on days 0, 4, 7, and 14.

### Micturition pattern detection

We adopted the widely used non-invasive method to detect the micturition pattern of model animals - Void spot assay (21). In simple terms, mice were placed in a circular transparent container with absorbent filter paper at the bottom, allowing mice to move and feed freely within 2 hours (daily from 9-11pm) but prohibiting water intake to avoid water stains interfering with the experimental results. Urine during the experiment will be retained by the absorbent filter paper, and the number of urination spots and the average urine volume per voiding of mice were evaluated by measuring using ImageJ software (22). Micturition pattern detection was performed on the 14th day.

### Quantitative real-time polymerase chain reaction

Total RNA was extracted following the manufacturer’s instructions with TRIzol reagent (Invitrogen, Carlsbad, CA, USA). To produce complementary DNA (cDNA), RNA was reverse transcribed using HiScript^®^ II Q RT SuperMix (Vazyme Biotech, Nanjing, Jiangsu, China). Quantitative real-time polymerase chain reaction (qRT-PCR) was done using 2 × RealStar Fast SYBR qPCR Mix (GenStar, Beijing, China) on a 7500 real-time PCR equipment (Applied Biosystems, Foster City, CA, USA).

β-Actin was used as an internal control gene. The primer sequences are listed in **Supplementary Table S1**.

### Enzyme-linked immunosorbent assay

After weighing, the bladder tissues were minced and mixed with a lysis buffer containing protease inhibitors. The mixture was thoroughly homogenized and lysed on ice. Subsequently, the homogenate was centrifuged at 4°C and 5,000 g for 10 minutes to remove precipitates, and the supernatant was collected. The concentration of CHI3L1 was measured using a Mouse CHI3L1 ELISA Kit (JL27055, Jianglai Biology, Shanghai) strictly following the manufacturer’s instructions. The absorbance was measured at 450 nm using a microplate reader. A standard curve was generated using the recombinant mouse CHI3L1 protein provided in the kit, and the CHI3L1 concentration in the samples was calculated based on the absorbance values.

### Cell culture

The RAW264.7 cells were generously provided by Professor Chang Chen. The culture system consists of DMEM-H+10% FBS + 1% Glutamax+1% Sodium Pyruvate+1% P/S. The atmosphere is 95% air+5% CO2, with a temperature of 37°C. The medium should be changed every 2–3 days, and passaging should be done at a ratio of 1:3-1:6.

### Construct stable overexpressing cell lines

In summary, a pBC03 expression vector was used to construct CHI3L1 overexpression plasmids and control group plasmids (**Supplementary Table S2**), which were transfected into RAW264.7 cells by electroporation. Screening was performed by adding selective antibiotic (Puromycin) to obtain stable RAW264.7 cell lines overexpressing CHI3L1.

### Assessment of mitochondrial respiratory function

The oxygen consumption rate (OCR) of cellular mitochondria was measured using a high-resolution respirometry system (O_2_k, Oroboros, Austria) (23). The procedure was briefly as follows: experimental cells were divided into two groups—control group (CHI3L1-NC) and CHI3L1 overexpression group (CHI3L1-OE). Cells were processed according to experimental requirements prior to detection. Cells in the logarithmic growth phase were harvested by trypsinization, washed, and resuspended in specialized assay medium. Accurate cell counting was performed using a hemocytometer, and the cell concentration of each sample was adjusted to 1 × 10^6^ cells/mL. Before starting measurements, the oxygen sensors were calibrated following the standard operational procedures of the O_2_k system to ensure accurate measurement of oxygen concentration within the chamber. A volume of 1 mL cell suspension was added to each of the two chambers. Under constant temperature conditions (37°C), specific inhibitors and uncouplers were sequentially injected to monitor basal respiration and the cellular respiratory response to different compounds. The OCR values (in pmol·s^-^¹) were recorded in real-time and calculated using the DatLab software coupled with the instrument. Key respiratory parameters were derived as follows: Basal Respiration: The last OCR measurement point before the injection of oligomycin. ATP-linked OCR: Calculated as basal respiration minus the stabilized OCR value after oligomycin injection. Maximal Respiration: The highest OCR value recorded after the injection of FCCP. Spare Respiratory Capacity: Determined as maximal respiration minus basal respiration, reflecting the functional reserve of the mitochondria. All experiments were independently repeated three times.

### Transcriptome sequencing and analysis

Use TRIzol reagent (Invitrogen, Carlsbad, CA, USA) to extract total RNA from cells, then use agarose gel to detect the quality of RNA. After ensuring the quality of RNA, send the RNA samples to Biomarker Technologies (Beijing, China) for library preparation and sequencing, generating raw sequencing data on the Illumina HiSeqTM 4000 platform. Subsequently, process the raw data by removing adapter sequences, poly-N sequences, and reads with poor quality. The cleaned data is aligned to the Mus musculus reference genome using HISAT2 tool to evaluate gene expression levels. Gene expression levels are estimated by calculating the coverage of each gene per kilobase of transcript per million mapped reads. Bioinformatics analysis was performed using BMKCloud (www.biocloud.net). If needed, original data can be requested from the corresponding author.

### Metabolomics sequencing and analysis

The metabolomics sequencing approach is consistent with our previous study (24). The study on RAW264.7 metabolomics utilized the UHPLC-Q-TOF method. The analysis was conducted using the Agilent 1290 II UPLC-QTOF 5600 PLUS liquid chromatography-mass spectrometry system. The ACQUITY UPLC HSS T3 column from Waters was employed, with a particle size of 1.8 μm and dimensions of 2.1 mm × 100 mm. The analysis was performed in the electric spray ionization (ESI) mode. Specific conditions and settings for the analysis included a curtain gas pressure of 35, an ion spray voltage of 5500 V in positive ion mode, -4500 V in negative ion mode, a temperature of 450°C, and ion source gas settings of 50 each for gas 1 and gas 2. The Agilent MassHunter workstation software (version B.01.04; Agilent, Lexington, MA, USA) was utilized to process the raw data. Isotope interference was removed, and noise was filtered out by setting the intensity threshold to 300. Metabolite identification was performed by referencing the METLIN open-source database (https://metlin.scripps.edu/landing_page.php?pgcontent=mainPage, access date: 11 October 2023). MetaboAnalyst 5.0 (http://www.metaboanalyst.ca/; Visited on October 12, 2021) was utilized for data preprocessing and bioinformatics analysis in this study (25). The group probability quotient normalization method was employed to normalize the data (26), with the NC group being the reference group. Log transformation (base 10) and the Pareto method (mean-centered and divided by the square root of the standard deviation of each variable) were used for data normalization. If needed, original data can be requested from the corresponding author.

### Statistical analysis

We performed statistical analysis using GraphPad Prism 8.0.1 software (GraphPad Software Inc., CA, USA) and R software 4.1.3 (Vienna, Austria). *In vivo* experiments included at least 6 biological replicates (n ≥ 6), and *in vitro* experiments included at least 3 technical replicates (n ≥ 3). The results of experiments are expressed as mean ± SD (standard deviation). When comparing the difference in means between two groups, use the Mann-Whitney U test when variances are unequal and the t-test when variances are equal. A statistically significant result was defined as *p* < 0.05.

## Results

### Integration analysis of IC/BPS transcriptome and clinical feature

Researchers in the field of urology worldwide have conducted extensive research on IC/BPS, mainly focusing on bladder tissues, blood samples, and urine samples for transcriptomics, metabolomics, microbiomics, or single-cell sequencing studies (16, 27–30), and most of the data have been shared with scholars worldwide. This provides a wealth of reliable reference materials for global scholars to collectively explore the mechanisms of IC/BPS. Therefore, we downloaded all currently available RNA-seq datasets of bladder tissues from IC/BPS patients and normal control groups from the GEO website, including GSE11783, GSE57560, and GSE621 (https://www.ncbi.nlm.nih.gov/geo/query/acc.cgi?acc=GSE11783, GSE57560, GSE621, accessed on August 1, 2023). The dataset GSE621, containing too few genes (N = 3353), was excluded from the study.

**Figure 1:**
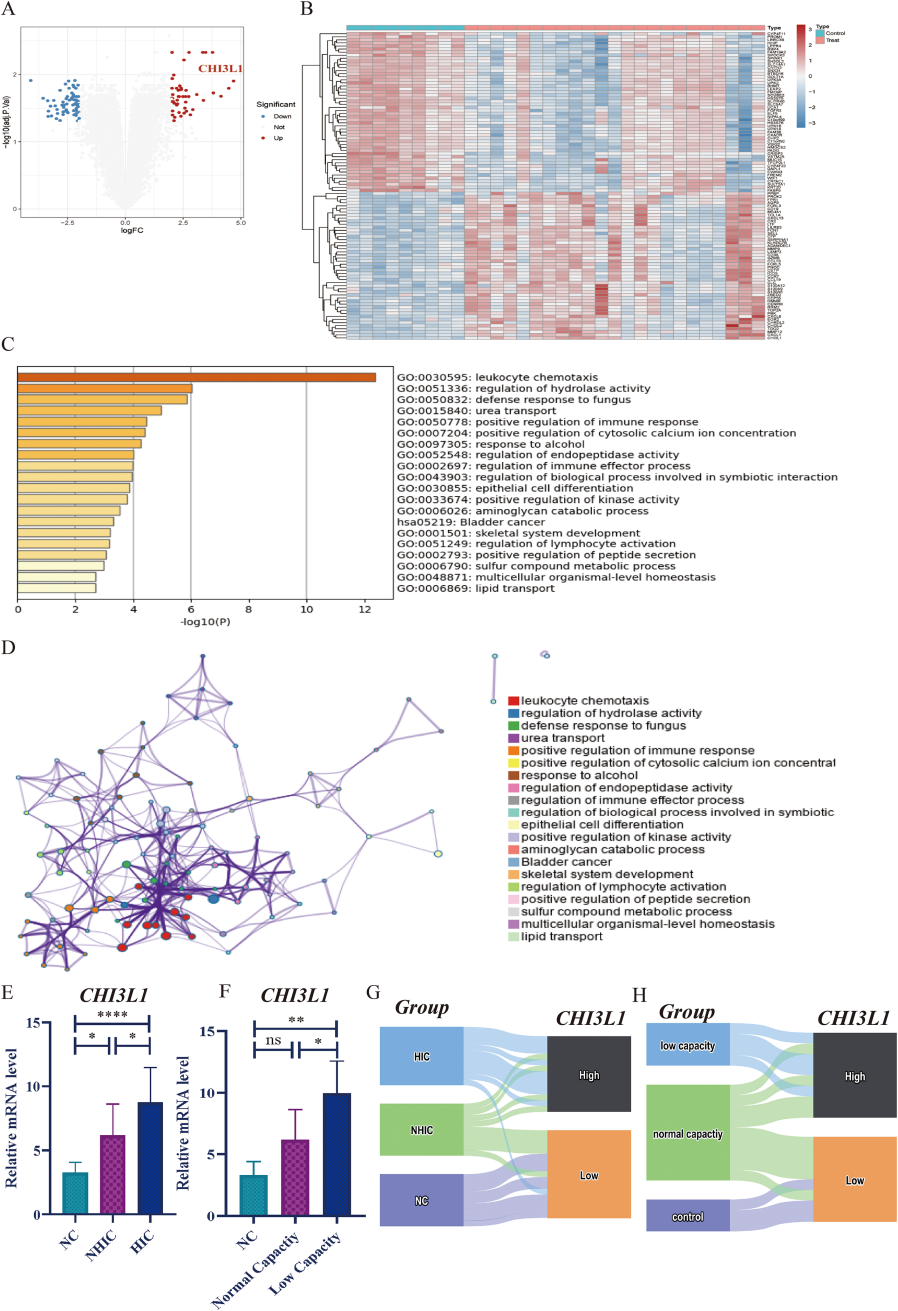
CHI3L1 is upregulated in HIC and correlates with clinical features. **(A, B)** Volcano plot and heatmap of differentially expressed genes (DEGs) in IC/BPS bladder tissues compared to normal controls. **(C, D)** Pathway enrichment analysis of DEGs, highlighting leukocyte chemotaxis as the most significantly enriched pathway. **(E)** CHI3L1 expression levels in normal controls, NHIC, and HIC patients. **(F)** Correlation between CHI3L1 expression and bladder capacity in IC/BPS patients. **(G, H)** Classification of patients into CHI3L1 high-expression and low-expression groups based on median expression value, showing association with HIC subtype and reduced bladder capacity. Statistical significance was determined and is displayed as follows: “ns” not significant , "*" p < 0.05, "* *" p < 0.01, and "* * * *" p < 0.0001.

The combined analysis of transcriptomic data allows us to have a more comprehensive understanding of what significant changes may have occurred in the bladders of IC/BPS patients. The integrated dataset includes 9 normal individuals and 23 patients with IC/BPS. We redefined the DEGs between the IC/BPS group and the normal control group (**Figures 1A, B**). Pathway enrichment analysis of DEGs helps us further understand what important changes have occurred in the bladder tissues of IC/BPS (**Figures 1C, D**). Among all pathways, leukocyte chemotaxis occupies the absolute leading position. The top twenty enriched pathways also include positive regulation of immune response, regulation of immune effector process, and regulation of lymphocyte. This suggests that PIEs are a key pathogenic mechanism of IC/BPS. To identify the most pivotal target genes involved in the PIEs process from a large number of candidates, we employed a multi-step screening strategy. The DEGs analysis, conducted with stringent statistical thresholds, revealed that Chitinase-3-like protein 1 (CHI3L1) exhibited the most significant upregulation with the highest fold change among all genes examined. The selection was further justified by CHI3L1’s established role in key IC/BPS pathologies like inflammation and fibrosis, making it a biologically plausible candidate, a finding subsequently validated by our preliminary experiments. Strategically, its nature as a secreted protein enhances its potential as a therapeutic target. The results above showed that CHI3L1 is the most promising target gene. It encodes a secreted glycoprotein mainly expressed in macrophages and neutrophils, and is currently considered to be closely related to inflammation and tissue remodeling processes (31). Researchers as early as 2010 discovered that CHI3L1 is upregulated in the serum and urine of IC/BPS patients, mainly expressed in detrusor mastocytes and submucosal macrophages, and is associated with detrusor fibrosis (32). Similarly, our current data also confirmed the association of CHI3L1 with IC/BPS features from two aspects. On the one hand, compared to the normal control group, the expression of CHI3L1 is significantly upregulated in patients with NHIC and HIC, with HIC subtype showing a significantly higher expression than NHIC subtype (**Figure 1E**). On the other hand, the expression of CHI3L1 is also associated with bladder capacity. In IC/BPS patients without a decrease in bladder capacity, their CHI3L1 expression is not significantly higher than that of the normal control group, but once the condition progresses to bladder contraction, CHI3L1 shows a significant upregulation (**Figure 1F**). Using the median value for CHI3L1 expression as the cutoff value, research study subjects were divided into high-expression and low-expression groups, with the HIC subtype and patients with reduced bladder capacity almost all classified as the high-expression group (**Figures 1G, H**). Combining the above findings, we speculate that the upregulation of CHI3L1 expression may be involved in the occurrence of PIEs in HIC patients, ultimately leading to bladder contractions.

### Validation of the expression status of CHI3L1 in the IC/BPS animal model

**Figure 2:**
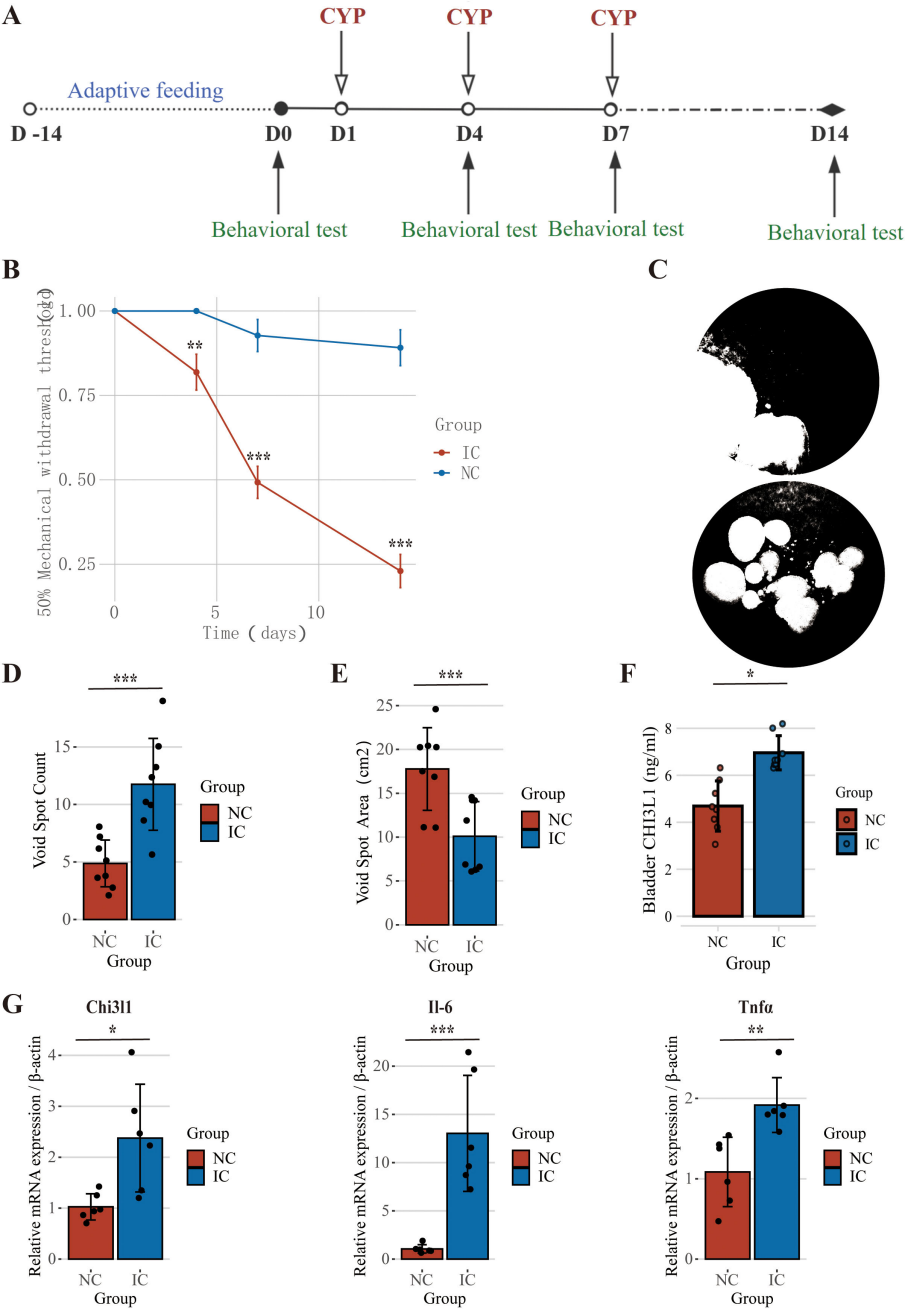
Validation of CHI3L1 upregulation in an IC/BPS mouse model. **(A)** Schematic of the cyclophosphamide-induced IC/BPS mouse model construction. **(B)** Mechanical pain threshold measurement using von Frey filaments. **(C–E)** Urination patterns showing increased frequency and reduced volume per void. **(F)** protein expression levels of CHI3L1 in bladder tissues of control and IC/BPS mice. **(G)** mRNA expression levels of CHI3L1, IL-6, and TNFα in bladder tissues of control and IC/BPS mice. Statistical significance was determined and is displayed as follows: "*" p < 0.05, "* *" p < 0.01, and "* * *" p < 0.001.

By using the IC/BPS mouse model, we preliminarily confirmed the expression trend of CHI3L1 in IC/BPS. The experimental methods were described in detail in the study, and specific steps were shown in **Figure 2A**. We observed that the IC/BPS mouse model exhibited a significant decrease in pain threshold (**Figure 2B**) and showed an increase in urination frequency and a decrease in average urination volume (**Figures 2C–E**) in terms of urination patterns. Finally, through the analysis of bladder tissues in the two groups, we found that the mRNA expression levels of the target gene.

CHI3L1 and the inflammation genes IL-6 and TNFα were significantly upregulated, and the protein expression of CHI3L1 in the IC group was also increased (**Figures 2F, G**). These results strongly support our successful use of the IC/BPS animal model to verify the upregulation of CHI3L1.

### The upregulation of CHI3L1 expression in macrophages is associated with IC/BPS related PIEs

**Figure 3:**
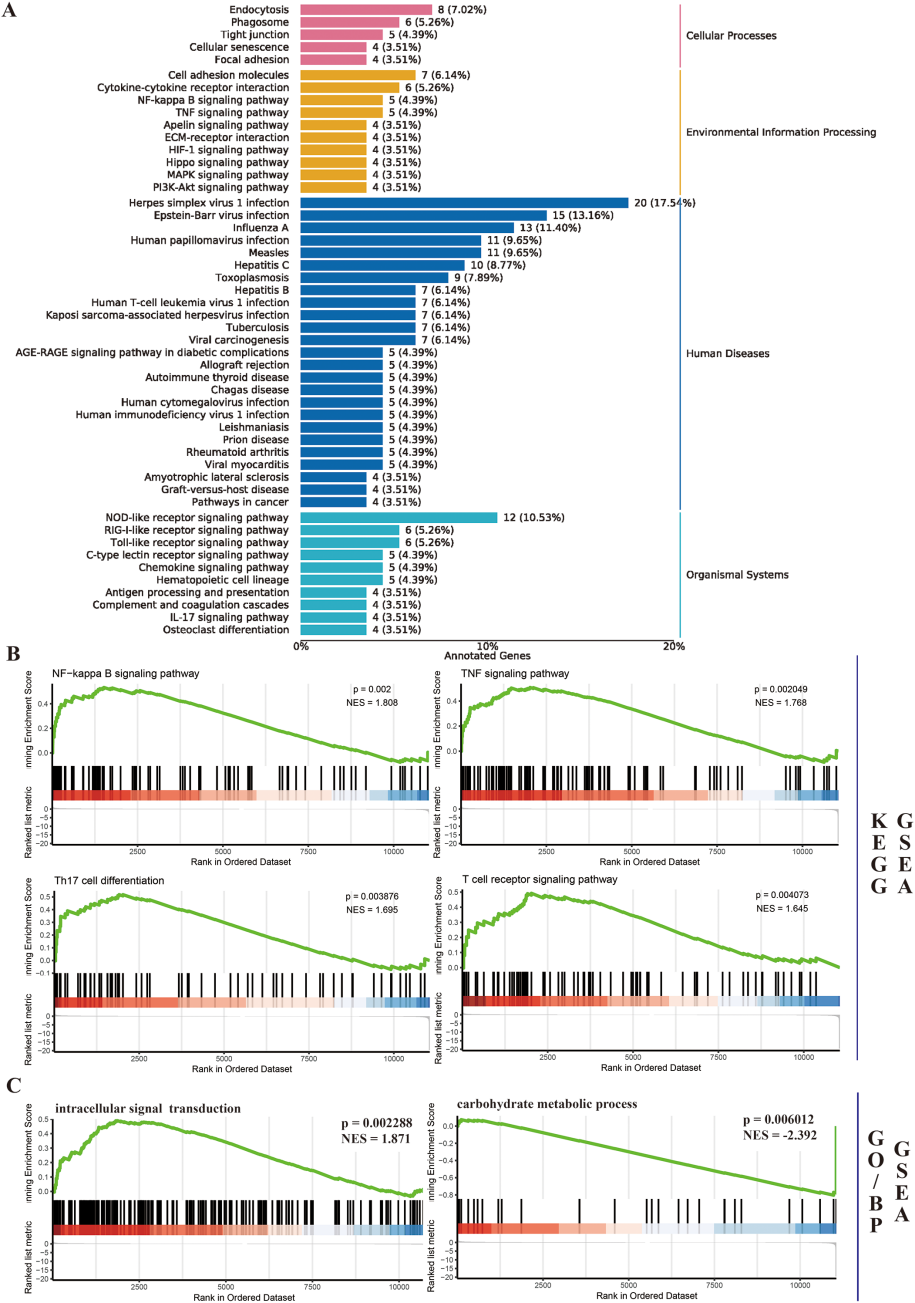
CHI3L1 overexpression in macrophages drives pro-inflammatory pathways. **(A)** KEGG analysis of DEGs in CHI3L1-overexpressing macrophages, categorized into Cellular Processes, Environmental Information Processing, Human Diseases, and Organismal Systems. **(B)** Gene Set Enrichment Analysis (GSEA) of upregulated (e.g., NF-κB, TNF signaling) and downregulated (e.g., virus-related) pathways. **(C)** Top enriched biological processes (BP) showing upregulation of intracellular signal transduction and T cell receptor pathways, and downregulation of carbohydrate metabolism and anti-infection processes.

Based on experimental findings demonstrating the coordinated upregulation of CHI3L1 and PIE markers in our cellular models, we hypothesize a correlation between PIE and CHI3L1 upregulation in the pathogenesis of IC/BPS. Previous studies have indicated that CHI3L1 is mainly expressed in mastocytes of the detrusor layer and submucosal macrophages (32). Our recent research results suggested that macrophages play an important role in the process of IC/BPS related PIEs (8, 16), so we further explored the regulatory effects and related mechanisms of CHI3L1 on macrophages. Firstly, we overexpressed CHI3L1 (mouse) in macrophages (RAW264.7), and then analyzed the changes in the macrophage gene expression profile through transcriptome sequencing. Using a fold change threshold of 1.5 and a p-value threshold of 0.05, a total of 219 differentially expressed genes were screened, including 136 upregulated and 83 downregulated genes. The KEGG analysis of differentially expressed genes is shown in **Figure 3A**. The enriched KEGG pathways are divided into four categories: Cellular processes, Environmental information processing, Human diseases, and Organismal Systems. Endocytosis and phagosome rank top in Cellular processes. In Environmental information processing, cell adhesion molecules, cytokine-cytokine receptor interaction, NF-kappa B signaling pathway, and TNF signaling pathway are also widely enriched. Most of the diseases in Human diseases are related to viruses. In Organismal Systems, there is enrichment of various receptor signaling pathways (NOD-like, RIG-I-like, and Toll-like receptor). Furthermore, we performed Gene Set Enrichment Analysis (GSEA) to analyze the upregulated and downregulated genes separately to further clarify the biological processes that CHI3L1 may affect. Among the top ten significantly upregulated KEGG pathways, seven are related to the reshaping of the inflammatory immune microenvironment (ko04064: NF-kappa B signaling pathway, ko04024: cAMP signaling pathway, ko04215: Apoptosis - multiple species, ko04668: TNF signaling pathway, ko04722: Neurotrophin signaling pathway, ko04659: Th17 cell differentiation, ko04660: T cell receptor signaling pathway), while the downregulated KEGG pathways are mostly virus-related pathways (**Figure 3B**). The most significant upregulated biological processes are intracellular signal transduction (GO:0035556) and T cell receptor signaling pathway (GO:0050852), and the most significant downregulated biological process is carbohydrate metabolic process (GO:0005975) (**Figure 3C**). In addition, important anti-infection processes of macrophages (GO:0042742: defense response to bacterium; GO:0051607: defense response to virus) and angiogenesis capability (GO:0045766: positive regulation of angiogenesis) are also among the most significantly downregulated BP lists. Based on the above findings, we conclude that the upregulation of CHI3L1 expression in macrophages promotes the activation of various inflammatory immune pathways, leading to the development of IC/BPS PIEs.

### The upregulation of CHI3L1 mediates macrophage activation

**Figure 4:**
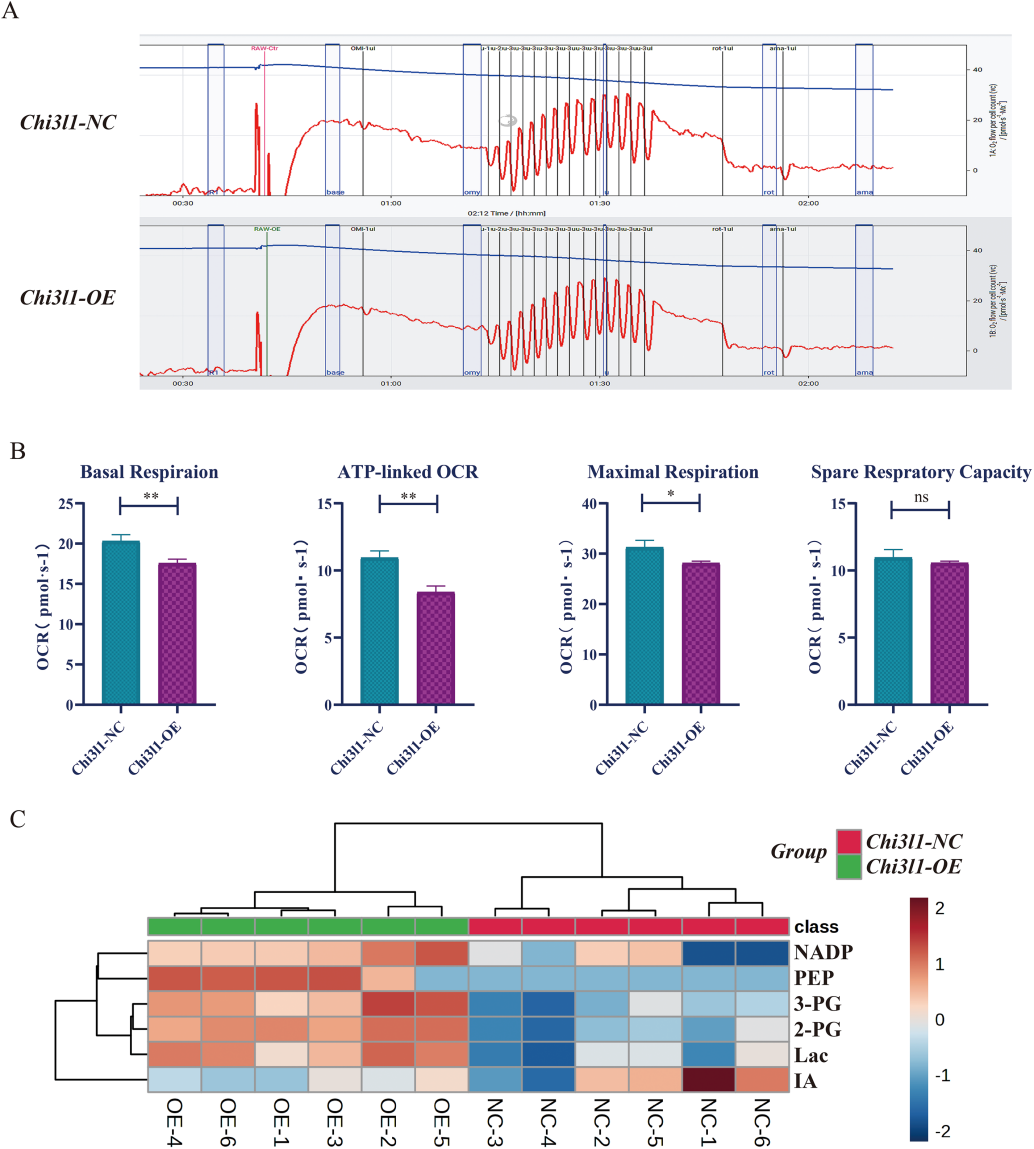
CHI3L1 reprograms macrophage metabolism toward glycolysis. **(A, B)** High-resolution respirometry (O2k) showing reduced basal respiration, maximal respiration, and ATP-linked OCR in CHI3L1-overexpressing macrophages. **(C)** Metabolomic analysis of glycolytic intermediates, showing upregulation of NADP, PEP, 3-PG, 2-PG, Lactate, and downregulation of Itaconic acid (IA). Statistical significance was determined and is displayed as follows: “ns” not significant, "*" p < 0.05, and "* *" p < 0.01.

As mentioned earlier, the energy metabolism of macrophages is an important mechanism for regulating their functional state (33). In order to explore the relationship between CHI3L1 and macrophage carbohydrate metabolic patterns, we used Oroboros’ O2k technology to measure the mitochondrial respiratory function of macrophages using high-resolution respirometry (**Figures 4A, B**). The basal respiration, maximal respiration, and ATP-linked OCR of the CHI3L1-OE group were decreased compared to the control group, suggesting mitochondrial OXPHOS dysfunction.

Metabolomic analysis of differential metabolites in the glycolytic metabolic pathway included upregulation of Nicotinic acid adenine dinucleotide phosphate (NADP), Phosphoenolpyruvic acid (PEP), 3-Phosphoglycerate (3-PG), 2-Phosphoglyceric acid (2-PG), and Lactate (Lac) in the CHI3L1-OE group, while Itaconic acid (IA) was downregulated (**Figure 4C**), the latter of which can inhibit M2 polarization of macrophages (34). The only DEGs related to glycolysis screened from the transcriptome was glyceraldehyde-3-phosphate dehydrogenase (GAPDH), which was significantly upregulated. GAPDH is the key enzyme in the sixth step of glycolysis, catalyzing the conversion of glyceraldehyde 3-phosphate to D-glycerate 1,3-diphosphate (35). The upregulation of GAPDH activity is one of the characteristics of macrophage activation (36), and inhibiting GAPDH activity can effectively reduce aerobic glycolysis, thereby exerting an anti-inflammatory effect. In conclusion, CHI3L1 enhances macrophage activation.

### CHI3L1 upregulation mediates inhibition of aspartate metabolism

**Figure 5:**
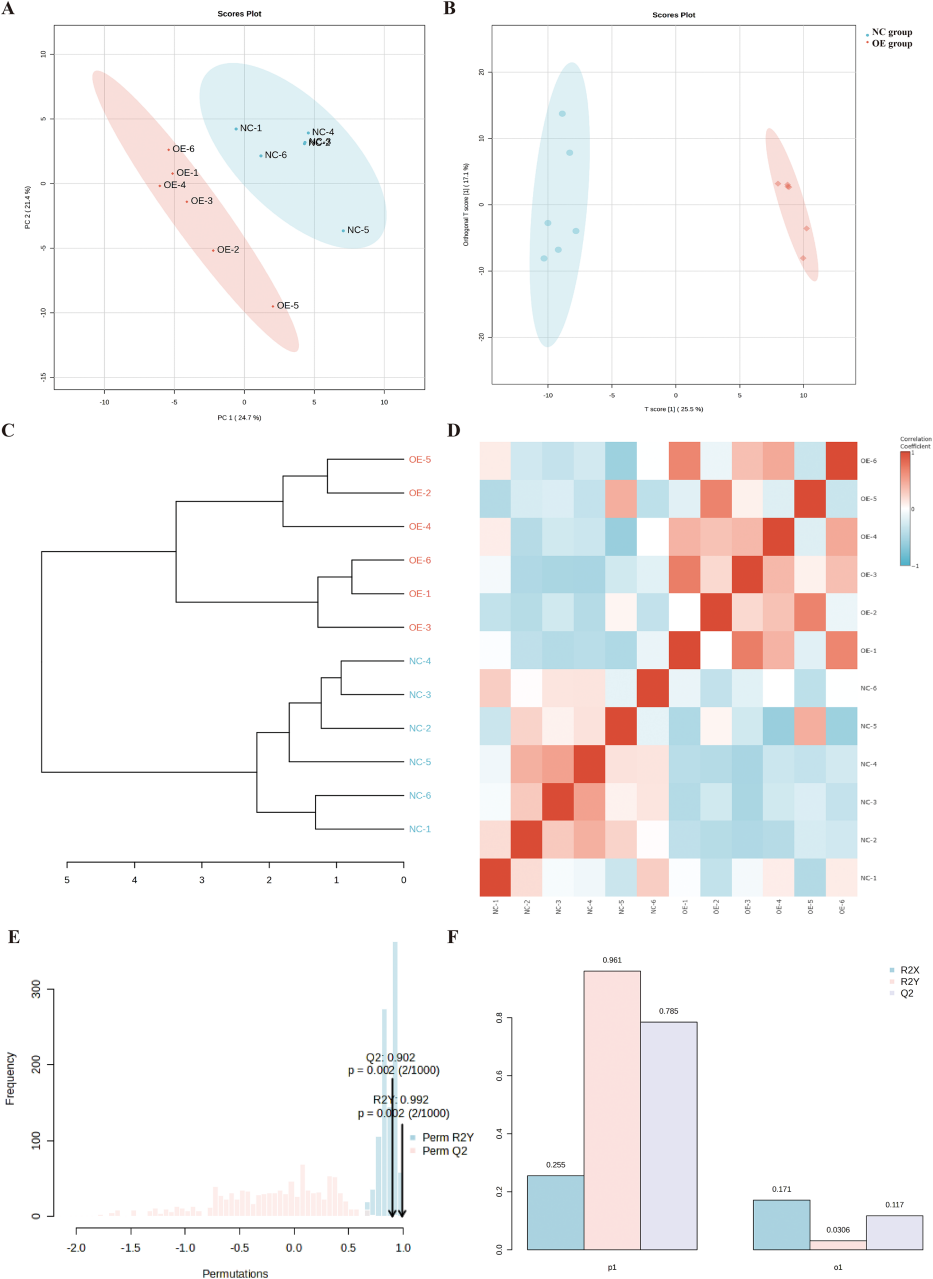
Metabolomic profiling reveals aspartate metabolism suppression by CHI3L1. **(A)** Principal Component Analysis (PCA) showing clear separation between control (NC) and CHI3L1-overexpression (OE) groups. **(B)** Orthogonal Partial Least Squares-Discriminant Analysis (OPLS-DA) confirming inter-group differences. **(C, D)** Pearson and Spearman correlation analyses demonstrating intra-group reproducibility and inter-group variability. **(E, F)** Permutation tests and cross-validation validating model stability without overfitting.

**Figure 6:**
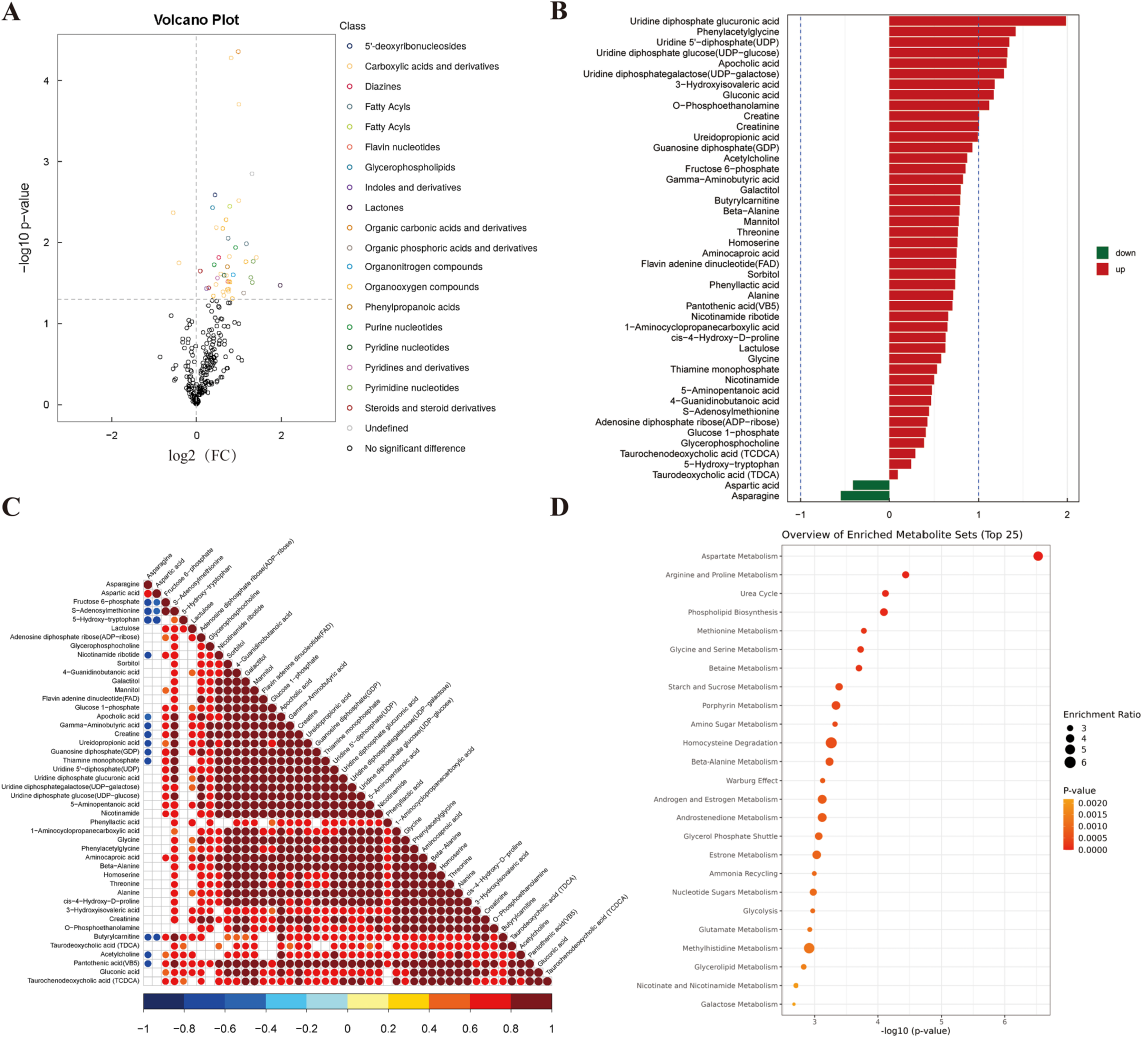
CHI3L1-mediated suppression of aspartate metabolism. **(A)** Volcanic plot of 46 differentially expressed metabolites between NC and OE groups. **(B)** Downregulation of aspartic acid and asparagine in the OE group. **(C)** Correlation network showing negative association between aspartate/asparagine and other metabolites. **(D)** Enrichment analysis identifying aspartate metabolism as the most altered pathway.

To further investigate the impact of expression on the metabolic profile of macrophages, we conducted targeted metabolomics analysis. A total of 362 distinct metabolites belonging to 38 subclasses were identified (**Supplementary Table S1**). Principal Component Analysis (PCA) demonstrated high intra-group consistency and clear inter-group separation, indicating the robustness of the metabolomic data (**Figure 5A**). Orthogonal Partial Least Squares-Discriminant Analysis (OPLS-DA) effectively revealed inter-group differences in the metabolomic profiles (**Figure 5B**). Both Pearson dendrogram clustering and Spearman correlation heatmap confirmed strong intra-group reproducibility within the NC and OE groups, along with significant inter-group variability, supporting the reliability of subsequent analyses (**Figures 5C, D**). Permutation tests and 5-fold cross-validation confirmed the excellent stability of the model without overfitting (**Figures 5E, F**). With a threshold of *p* < 0.05, 46 differentially expressed metabolites were identified between the two groups, most of which were upregulated in the OE group (**Figure 6A**). Only aspartic acid and asparagine showed decreased expression in the OE group (**Figure 6B**). Correlation analysis revealed that aspartic acid and asparagine were positively correlated with each other but negatively correlated with most other differentially expressed metabolites (**Figure 6C**). Enrichment analysis of all differential metabolites further confirmed that aspartate metabolism was the most significantly altered pathway (**Figure 6D**).

### CHI3L1 is expressed in macrophages and shows a significant positive correlation with MYC

**Figure 7:**
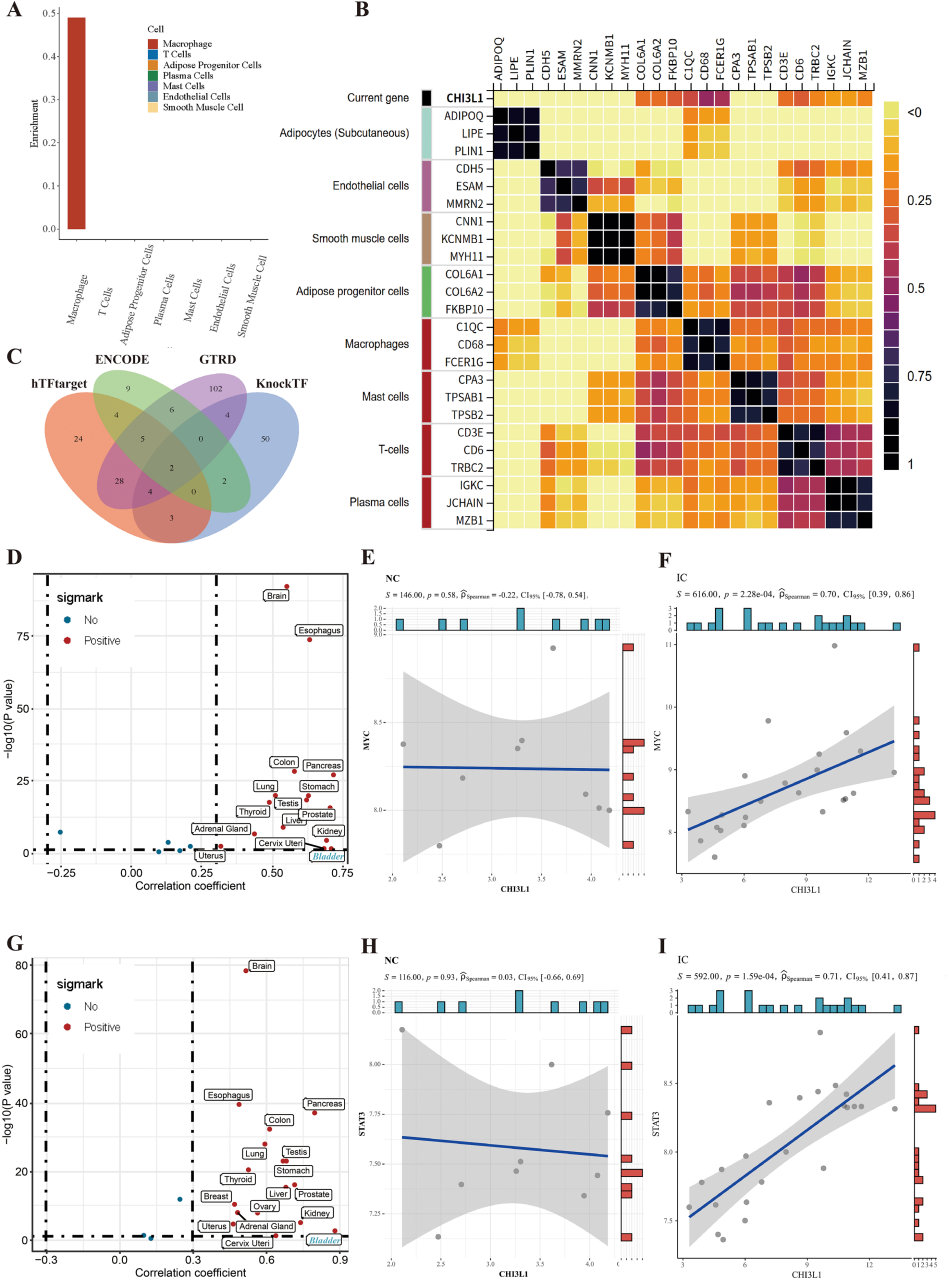
CHI3L1 correlates with MYC in macrophages under inflammatory conditions. **(A)** Human Protein Atlas data showing CHI3L1 expression in macrophages of visceral adipose tissue. **(B)** Correlation between CHI3L1 and macrophage marker genes (C1QC, CD68, FCER1G). **(C)** Identification of MYC and STAT3 as common transcription factors downstream of CHI3L1 across four databases. **(D-I)** Correlation analysis of CHI3L1 with MYC and STAT3 in normal human tissues (GTEx) and IC/BPS datasets, showing strong positive correlation with MYC in inflammatory contexts.

Utilizing THE HUMAN PROTEIN ATLAS database, we investigated the tissue distribution of human CHI3L1. Although data on bladder tissue were not available in the database, we found the most prominent evidence of CHI3L1 expression in macrophages within similar visceral adipose tissue (**Figure 7A**). Similarly, CHI3L1 expression exhibited the highest correlation coefficients (ranging from 0.33 to 0.458) with three macrophage marker genes (C1QC, CD68, and FCER1G) (**Figure 7B**). To explore potential transcription factors through which CHI3L1 might mediate functional changes in macrophages, we employed the TFTF tool (37) to integrate four major transcription factor databases (hTFtarget, KnockTF, ENCODE, and GTRD) for identifying downstream transcription factors of CHI3L1. Two common transcription factors, MYC and STAT3, were identified across all four databases (**Figure 7C**). We then analyzed the correlation between CHI3L1 expression and MYC and STAT3 in normal human tissue samples from the Genotype-Tissue Expression (GTEx) project and within our integrated dataset (**Figures 7D–I**). **Figures 7D, E** revealed a significant positive correlation between CHI3L1 and both MYC and STAT3 in bladder tissue. This correlation nearly disappeared in the normal control (NC) group (**Figures 7E, H**) but was further validated in the experimental or intervention (IC) group (**Figures 7F, I**). Previous studies have demonstrated that MYC promotes M1 polarization of macrophages (38, 39) and enhances the glycolytic process (40, 41), while STAT3 plays a key role in M2 polarization of macrophages, facilitating anti-inflammatory and immunoregulatory functions (42, 43). In summary, we speculate that CHI3L1-mediated macrophage activation under specific conditions (e.g., in HIC) may be associated with MYC.

## Discussion

**Figure 8:**
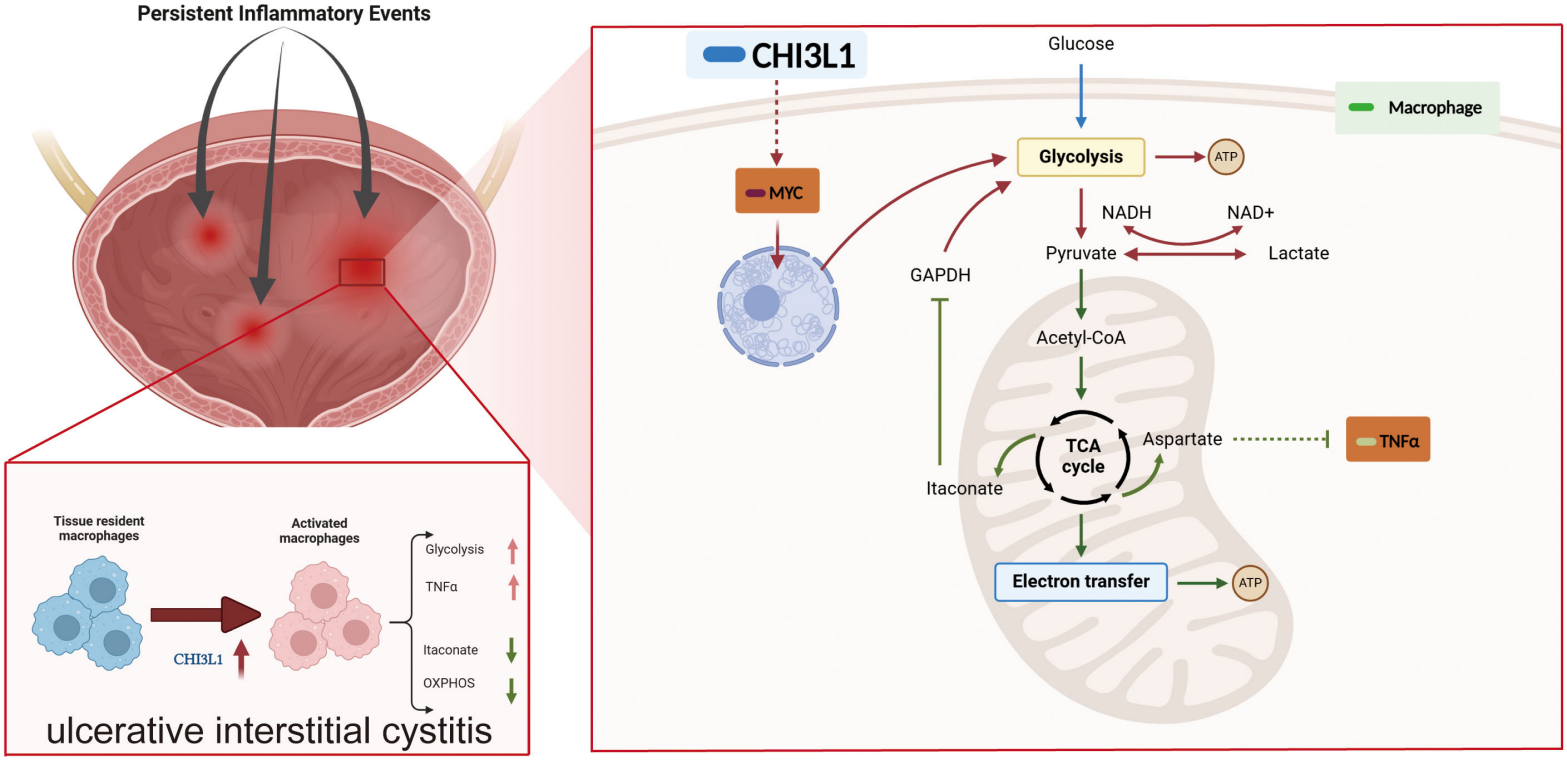
CHI3L1 exacerbates the pathogenesis of interstitial cystitis (IC) by reprogramming macrophage metabolism and promoting a pro-inflammatory phenotype.

This study investigated the pathogenesis of HIC through integrated bioinformatic analysis, cellular experiments, and animal models. Our findings demonstrate that CHI3L1 expression is significantly upregulated in HIC patients and is closely associated with reduced bladder capacity. Overexpression of CHI3L1 in macrophages drives a pro-inflammatory phenotype by activating key signaling pathways such as NF-κB and TNFa, and by promoting glycolysis. CHI3L1-mediated activation suppresses mitochondrial OXPHOS and disrupts aspartate metabolism. Furthermore, under inflammatory conditions, CHI3L1 expression strongly correlates with the oncogenic transcription factor MYC rather than STAT3. These results reveal a novel mechanism by which CHI3L1 exacerbates PIEs in HIC through metabolic reprogramming and enhanced activation of macrophages (**Figure 8**).

The significant upregulation of CHI3L1 in HIC patients, particularly those with diminished bladder capacity, aligns with its established role as a biomarker of severe inflammation and tissue remodeling in various chronic conditions (31). This observation also shares potential similarities with the increase of CHI3L1 seen in liver fibrosis (44). Our integrated transcriptomic analysis not only confirmed the involvement of immune processes—such as leukocyte chemotaxis—but also specifically highlighted the central role of CHI3L1. Elevated levels of CHI3L1 have been widely reported in numerous inflammatory and neoplastic diseases (45). Recent studies further reveal that CHI3L1 exerts multiple immunomodulatory effects, including suppression of innate immune responses, stimulation of immune checkpoint molecules, and inhibition of T-cell costimulation, suggesting that its immunoregulatory functions may extend far beyond the bladder microenvironment (46).

A key mechanistic insight of this study is the profound impact of CHI3L1 on macrophage metabolism. The observed suppression of mitochondrial OXPHOS, coupled with upregulation of glycolytic intermediates (e.g., PEP, 3-PG, 2-PG, Lac) and the key glycolytic enzyme GAPDH, indicates a metabolic shift toward aerobic glycolysis. This reprogramming, known as the Warburg effect, is an established driver of M1-like pro-inflammatory macrophage activation (47). Itaconic acid, which is upregulated in M2 macrophages and exerts anti-inflammatory effects by inhibiting glycolysis through post-translational modification of GAPDH, was downregulated in our model, further reinforcing the CHI3L1-induced pro-inflammatory milieu (48–51).

Transcriptomic analysis revealed that CHI3L1 overexpression was accompanied by increased expression of TNFA, a representative marker of IC/BPS. Metabolomic profiling further demonstrated significant suppression of aspartate metabolism following CHI3L1 overexpression. These phenomena may be interrelated: studies in rheumatoid arthritis have shown that mitochondrial aspartate deficiency promotes TNFα synthesis (52). Aspartate is a key metabolite for protein synthesis, nucleotide production, and the malate-aspartate shuttle, which is essential for efficient mitochondrial respiration. Its depletion likely contributes directly to OXPHOS dysfunction and forces greater cellular reliance on glycolysis (53). This finding adds a new dimension to the understanding of how CHI3L1 reprograms cellular metabolism to foster a pro-inflammatory state.

The transcription factor c-Myc (MYC), encoded by the Myc oncogene, plays important roles in altering cellular metabolic pathways, cell growth, and proliferation. The strong positive correlation between CHI3L1 and MYC—but not STAT3—in the inflammatory context of HIC provides a plausible mechanism for sustaining the M1-like pro-inflammatory phenotype, given that MYC is a master regulator of glycolysis (54, 55). However, the precise molecular mechanisms by which CHI3L1 regulates MYC activity remain unclear and may involve binding to known receptors (e.g., IL-13Rα2), subsequently activating downstream signaling cascades (e.g., MAPK pathways) that converge on MYC stabilization or transcriptional control (56, 57).

This study has several limitations that should be considered. The primary limitation stems from the *in vitro* model system. The use of RAW264.7 cells cultured in DMEM-H medium containing 25 mM glucose, while a standard protocol for maintaining cell viability, creates a supraphysiological metabolic environment. The observed downregulation of the broader carbohydrate metabolic process may therefore be specific to this high-glucose condition and may not fully recapitulate the *in vivo* pathology of IC/BPS, especially in light of clinical evidence indicating that such downregulation is not a hallmark in IC/BPS patient samples (58, 59). Furthermore, the speculative interpretation regarding the coexistence of GAPDH upregulation and carbohydrate metabolite downregulation requires caution. The findings from this specific cell culture model necessitate further validation in more pathologically relevant systems, such as primary cells cultured at physiological glucose levels or animal models of IC/BPS, to confirm their biological and clinical significance.

The translational implications of our findings are constrained by several methodological limitations. First, the main functional validation was performed in a murine macrophage cell line (RAW264.7), which may not fully recapitulate the behavior of human primary macrophages or the complex *in vivo* microenvironment of the human bladder in HIC. Second, our *in vivo* data rely on a cyclophosphamide-induced IC/BPS mouse model. Although this model exhibits key features of bladder inflammation and pain, it may not fully mirror the chronicity and heterogeneity of human HIC, particularly specific immune cell interactions and stromal contributions. Besides, while this model has been valuable for elucidating the role of CHI3L1 in this study, it is important to note that it may not fully represent the entire spectrum of IC/BPS in humans. Future studies employing alternative or complementary animal models would be beneficial to further validate and extend our findings. Third, although we identified a correlation between CHI3L1 and MYC, our study did not provide direct genetic evidence (e.g., MYC knockdown) to establish causality in the observed macrophage activation and metabolic changes. Future studies using conditional knockout models or pharmacological inhibitors targeting MYC in a macrophage-specific manner are essential to validate this proposed pathway. Furthermore, as suggested by the findings of Liu et al. and Inal-Gultekin et al. (60, 61), the pathogenesis of IC/BPS may involve various adhesion molecules beyond CHI3L1, so we fully acknowledge the potential roles of other DEGs and have noted them as fruitful avenues for future investigation. As a final point, we propose that the diagnostic utility of CHI3L1 might be optimized by combining it with other HIC-specific markers (e.g., specific urinary cytokines or histopathological characteristics) into a multi-analyte panel, which represents a key objective for future investigation.

Notwithstanding these limitations, our findings position CHI3L1 as a promising therapeutic target in HIC. Its central role in perpetuating inflammation and metabolic dysfunction suggests that inhibiting CHI3L1 could disrupt the cycle of chronic inflammation. Emerging strategies include monoclonal antibodies against CHI3L1 or small-molecule inhibitors disrupting its interaction with receptors. However, potential on-target side effects must be carefully considered due to the pleiotropic functions of CHI3L1 in tissue repair and infection response. For example, CHI3L1 deficiency has been shown to exacerbate acute inflammation or impair pathogen clearance in some contexts (62). Therefore, future research should explore bladder-specific targeting strategies to maximize therapeutic efficacy while minimizing systemic adverse effects.

## Data Availability

The original contributions presented in the study are publicly available. This data can be found here: https://doi.org/10.6084/m9.figshare.30265135.v1.

## Glossary

IC/BPS: interstitial cystitis/bladder pain syndrome
HIC: Hunner-type interstitial cystitis
NHIC: non-Hunner type interstitial cystitis
PIEs: persistent inflammatory events
CHI3L1: Chitinase-3-like protein 1
DEGs: differentially expressed genes
GEO: Gene Expression Omnibus
KEGG: Kyoto Encyclopedia of Genes and Genomes
GSEA: Gene Set Enrichment Analysis
OXPHOS: oxidative phosphorylation
OCR: oxygen consumption rate
PCA: Principal Component Analysis
OPLS-DA: Orthogonal Partial Least Squares-Discriminant Analysis
MYC: myelocytomatosis oncogene
STAT3: Signal Transducer and Activator of Transcription 3
NF-κB: Nuclear Factor-kappa B
TNF: Tumor Necrosis Factor
IL-6: Interleukin-6
TNFα: Tumor Necrosis Factor alpha
GAPDH: glyceraldehyde-3-phosphate dehydrogenase
NADP: Nicotinic acid adenine dinucleotide phosphate
PEP: Phosphoenolpyruvic acid
3-PG: 3-Phosphoglycerate
2-PG: 2-Phosphoglyceric acid
Lac: Lactate
IA: Itaconic acid
qRT-PCR: quantitative Real-Time Polymerase Chain Reaction
cDNA: complementary DNA
FBS: fetal bovine serum
DMEM: Dulbecco’s Modified Eagle Medium
P/S: Penicillin-Streptomycin
SD: standard deviation

## References

[1] Clemens JQ Erickson DR Varela NP Lai HH . Diagnosis and treatment of interstitial cystitis/bladder pain syndrome. J urology. (2022) 208:34–42. doi: 10.1097/JU.0000000000002756, PMID: 35536143

[2] van de Merwe JP Nordling J Bouchelouche P Bouchelouche K Cervigni M Daha LK . Diagnostic criteria, classification, and nomenclature for painful bladder syndrome/interstitial cystitis: an ESSIC proposal. Eur urology. (2008) 53:60–7. doi: 10.1016/j.eururo.2007.09.019, PMID: 17900797

[3] Akiyama Y Maeda D Katoh H Morikawa T Niimi A Nomiya A . Molecular taxonomy of interstitial cystitis/bladder pain syndrome based on whole transcriptome profiling by next-generation RNA sequencing of bladder mucosal biopsies. J urology. (2019) 202:290–300. doi: 10.1097/JU.0000000000000234, PMID: 30865573

[4] Blalock EM Korrect GS Stromberg AJ Erickson DR . Gene expression analysis of urine sediment: evaluation for potential noninvasive markers of interstitial cystitis/bladder pain syndrome. J urology. (2012) 187:725–32. doi: 10.1016/j.juro.2011.09.142, PMID: 22177197

[5] Chennamsetty A Khourdaji I Goike J Killinger KA Girdler B Peters KM . Electrosurgical management of Hunner ulcers in a referral center's interstitial cystitis population. Urology. (2015) 85:74–8. doi: 10.1016/j.urology.2014.09.012, PMID: 25440759

[6] Funaro MG King AN Stern JNH Moldwin RM Bahlani S . Endoscopic injection of low dose triamcinolone: A simple, minimally invasive, and effective therapy for interstitial cystitis with hunner lesions. Urology. (2018) 118:25–9. doi: 10.1016/j.urology.2018.03.037, PMID: 29782887

[7] Forrest JB Payne CK Erickson DR . Cyclosporine A for refractory interstitial cystitis/bladder pain syndrome: experience of 3 tertiary centers. J urology. (2012) 188:1186–91. doi: 10.1016/j.juro.2012.06.023, PMID: 22901569

[8] Zhang W Liu X Wang J Wang X Zhang Y . Immunogenic cell death associated molecular patterns and the dual role of IL17RA in interstitial cystitis/bladder pain syndrome. Biomolecules. (2023) 13:421. doi: 10.3390/biom13030421, PMID: 36979355 PMC10046465

[9] Gomez Perdiguero E Klapproth K Schulz C Busch K Azzoni E Crozet L . Tissue-resident macrophages originate from yolk-sac-derived erythro-myeloid progenitors. Nature. (2015) 518:547–51. doi: 10.1038/nature13989, PMID: 25470051 PMC5997177

[10] Blériot C Chakarov S Ginhoux F . Determinants of resident tissue macrophage identity and function. Immunity. (2020) 52:957–70. doi: 10.1016/j.immuni.2020.05.014, PMID: 32553181

[11] Tannahill GM Curtis AM Adamik J Palsson-McDermott EM McGettrick AF Goel G . Succinate is an inflammatory signal that induces IL-1β through HIF-1α. Nature. (2013) 496:238–42. doi: 10.1038/nature11986, PMID: 23535595 PMC4031686

[12] Mora-Bau G Platt AM van Rooijen N Randolph GJ Albert ML Ingersoll MA . Macrophages subvert adaptive immunity to urinary tract infection. PloS pathogens. (2015) 11:e1005044. doi: 10.1371/journal.ppat.1005044, PMID: 26182347 PMC4504509

[13] Ryan DG Murphy MP Frezza C Prag HA Chouchani ET O'Neill LA . Coupling Krebs cycle metabolites to signalling in immunity and cancer. Nat Metab. (2019) 1:16–33. doi: 10.1038/s42255-018-0014-7, PMID: 31032474 PMC6485344

[14] O'Neill LA Kishton RJ Rathmell J . A guide to immunometabolism for immunologists. Nat Rev Immunol. (2016) 16:553–65. doi: 10.1038/nri.2016.70, PMID: 27396447 PMC5001910

[15] Vijayan V Pradhan P Braud L Fuchs HR Gueler F Motterlini R . Human and murine macrophages exhibit differential metabolic responses to lipopolysaccharide - A divergent role for glycolysis. Redox Biol. (2019) 22:101147. doi: 10.1016/j.redox.2019.101147, PMID: 30825774 PMC6396203

[16] Su F Zhang W Meng L Zhang W Liu X Liu X . Multimodal single-cell analyses outline the immune microenvironment and therapeutic effectors of interstitial cystitis/bladder pain syndrome. Advanced Sci (Weinheim Baden-Wurttemberg Germany). (2022) 9:e2106063. doi: 10.1002/advs.202106063, PMID: 35470584 PMC9218658

[17] Zhou Y Zhou B Pache L Chang M Khodabakhshi AH Tanaseichuk O . Metascape provides a biologist-oriented resource for the analysis of systems-level datasets. Nat Commun. (2019) 10:1523. doi: 10.1038/s41467-019-09234-6, PMID: 30944313 PMC6447622

[18] Bon K Lichtensteiger CA Wilson SG Mogil J . Characterization of cyclophosphamide cystitis, a model of visceral and referred pain, in the mouse: species and strain differences. J urology. (2003) 170:1008–12. doi: 10.1097/01.ju.0000079766.49550.94, PMID: 12913760

[19] Ding H Chen J Su M Lin Z Zhan H Yang F . BDNF promotes activation of astrocytes and microglia contributing to neuroinflammation and mechanical allodynia in cyclophosphamide-induced cystitis. J neuroinflammation. (2020) 17:19. doi: 10.1186/s12974-020-1704-0, PMID: 31931832 PMC6958761

[20] Christensen SL Hansen RB Storm MA Olesen J Hansen TF Ossipov M . Von Frey testing revisited: Provision of an online algorithm for improved accuracy of 50% thresholds. Eur J Pain (London England). (2020) 24:783–90. doi: 10.1002/ejp.1528, PMID: 31889375

[21] Hill WG Zeidel ML Bjorling DE Vezina CM . Void spot assay: recommendations on the use of a simple micturition assay for mice. Am J Physiol Renal Physiol. (2018) 315:F1422–f9. doi: 10.1152/ajprenal.00350.2018, PMID: 30156116 PMC6293303

[22] Wegner KA Abler LL Oakes SR Mehta GS Ritter KE Hill WG . Void spot assay procedural optimization and software for rapid and objective quantification of rodent voiding function, including overlapping urine spots. Am J Physiol Renal Physiol. (2018) 315:F1067–f80. doi: 10.1152/ajprenal.00245.2018, PMID: 29972322 PMC6230749

[23] Ferreira JJ Cassina A Irigoyen P Ford M Pietroroia S Peramsetty N . Increased mitochondrial activity upon CatSper channel activation is required for mouse sperm capacitation. Redox Biol. (2021) 48:102176. doi: 10.1016/j.redox.2021.102176, PMID: 34753004 PMC8585656

[24] Zhang W Yang Q Song Y Liu W Li Y . Exploratory metabolomic analysis for characterizing the metabolic profile of the urinary bladder under estrogen deprivation. Front endocrinology. (2024) 15:1384115. doi: 10.3389/fendo.2024.1384115, PMID: 38883607 PMC11176512

[25] Pang Z Chong J Zhou G de Lima Morais DA Chang L Barrette M . MetaboAnalyst 5.0: narrowing the gap between raw spectra and functional insights. Nucleic Acids Res. (2021) 49:W388–w96. doi: 10.1093/nar/gkab382, PMID: 34019663 PMC8265181

[26] Dieterle F Ross A Schlotterbeck G Senn H . Probabilistic quotient normalization as robust method to account for dilution of complex biological mixtures. Appl 1H NMR metabonomics Analytical Chem. (2006) 78:4281–90. doi: 10.1021/ac051632c, PMID: 16808434

[27] Walton I Nickel JC . The urinary microbiome in interstitial cystitis/bladder pain syndrome? A critical look at the evidence. J urology. (2021) 206:1087–90. doi: 10.1097/JU.0000000000001947, PMID: 34184928

[28] Moldwin RM Nursey V Yaskiv O Dalvi S Macdonald EJ Funaro M . Immune cell profiles of patients with interstitial cystitis/bladder pain syndrome. J Trans Med. (2022) 20:97. doi: 10.1186/s12967-022-03236-7, PMID: 35193610 PMC8862517

[29] Logadottir Y Delbro D Fall M Gjertsson I Jirholt P Lindholm C . Cytokine expression in patients with bladder pain syndrome/interstitial cystitis ESSIC type 3C. J urology. (2014) 192:1564–8. doi: 10.1016/j.juro.2014.04.099, PMID: 24813342

[30] Colaco M Koslov DS Keys T Evans RJ Badlani GH Andersson KE . Correlation of gene expression with bladder capacity in interstitial cystitis/bladder pain syndrome. J urology. (2014) 192:1123–9. doi: 10.1016/j.juro.2014.05.047, PMID: 24840534

[31] Huang H Wu T Mao J Fang Y Zhang J Wu L . CHI3L1 is a liver-enriched, noninvasive biomarker that can be used to stage and diagnose substantial hepatic fibrosis. Omics: J Integr Biol. (2015) 19:339–45. doi: 10.1089/omi.2015.0037, PMID: 26415140 PMC4486713

[32] Richter B Roslind A Hesse U Nordling J Johansen JS Horn T . YKL-40 and mast cells are associated with detrusor fibrosis in patients diagnosed with bladder pain syndrome/interstitial cystitis according to the 2008 criteria of the European Society for the Study of Interstitial Cystitis. Histopathology. (2010) 57:371–83. doi: 10.1111/j.1365-2559.2010.03640.x, PMID: 20840668

[33] Liu Y Xu R Gu H Zhang E Qu J Cao W . Metabolic reprogramming in macrophage responses. biomark Res. (2021) 9:1. doi: 10.1186/s40364-020-00251-y, PMID: 33407885 PMC7786975

[34] O'Neill LAJ Artyomov MN . Itaconate: the poster child of metabolic reprogramming in macrophage function. Nat Rev Immunol. (2019) 19:273–81. doi: 10.1038/s41577-019-0128-5, PMID: 30705422

[35] Colell A Green DR Ricci JE . Novel roles for GAPDH in cell death and carcinogenesis. Cell Death differentiation. (2009) 16:1573–81. doi: 10.1038/cdd.2009.137, PMID: 19779498

[36] Kornberg MD Bhargava P Kim PM Putluri V Snowman AM Putluri N . Dimethyl fumarate targets GAPDH and aerobic glycolysis to modulate immunity. Sci (New York NY). (2018) 360:449–53. doi: 10.1126/science.aan4665, PMID: 29599194 PMC5924419

[37] Wang J . TFTF: an R-based integrative tool for decoding human transcription factor-target interactions. Biomolecules. (2024) 14:749. doi: 10.3390/biom14070749, PMID: 39062464 PMC11274450

[38] Esser AK Ross MH Fontana F Su X Gabay A Fox GC . Nanotherapy delivery of c-myc inhibitor targets Protumor Macrophages and preserves Antitumor Macrophages in Breast Cancer. Theranostics. (2020) 10:7510–26. doi: 10.7150/thno.44523, PMID: 32685002 PMC7359087

[39] Zhang Y Zhang C Feng R Meng T Peng W Song J . CXCR4 regulates macrophage M1 polarization by altering glycolysis to promote prostate fibrosis. Cell communication signaling: CCS. (2024) 22:456. doi: 10.1186/s12964-024-01828-y, PMID: 39327570 PMC11426013

[40] Lin J Wang X Zhai S Shi M Peng C Deng X . Hypoxia-induced exosomal circPDK1 promotes pancreatic cancer glycolysis via c-myc activation by modulating miR-628-3p/BPTF axis and degrading BIN1. J Hematol Oncol. (2022) 15:128. doi: 10.1186/s13045-022-01348-7, PMID: 36068586 PMC9450374

[41] Li M Yu J Ju L Wang Y Jin W Zhang R . USP43 stabilizes c-Myc to promote glycolysis and metastasis in bladder cancer. Cell Death disease. (2024) 15:44. doi: 10.1038/s41419-024-06446-7, PMID: 38218970 PMC10787741

[42] Xu J Zhang J Zhang Z Gao Z Qi Y Qiu W . Hypoxic glioma-derived exosomes promote M2-like macrophage polarization by enhancing autophagy induction. Cell Death disease. (2021) 12:373. doi: 10.1038/s41419-021-03664-1, PMID: 33828078 PMC8026615

[43] Xia T Zhang M Lei W Yang R Fu S Fan Z . Advances in the role of STAT3 in macrophage polarization. Front Immunol. (2023) 14:1160719. doi: 10.3389/fimmu.2023.1160719, PMID: 37081874 PMC10110879

[44] Nishimura N De Battista D McGivern DR Engle RE Tice A Fares-Gusmao R . Chitinase 3-like 1 is a profibrogenic factor overexpressed in the aging liver and in patients with liver cirrhosis. Proc Natl Acad Sci United States America. (2021) 118:e2019633118. doi: 10.1073/pnas.2019633118, PMID: 33888584 PMC8092404

[45] Zhao T Su Z Li Y Zhang X You Q . Chitinase-3 like-protein-1 function and its role in diseases. Signal transduction targeted Ther. (2020) 5:201. doi: 10.1038/s41392-020-00303-7, PMID: 32929074 PMC7490424

[46] Ma B Kamle S Sadanaga T Lee CM Lee JH Yee DC . Chitinase 3-like-1 inhibits innate antitumor and tissue remodeling immune responses by regulating CD47-SIRPα- and CD24-siglec10-mediated phagocytosis. J Immunol (Baltimore Md: 1950). (2024) 213:1279–91. doi: 10.4049/jimmunol.2400035, PMID: 39291933 PMC12422026

[47] Zhang D Tang Z Huang H Zhou G Cui C Weng Y . Metabolic regulation of gene expression by histone lactylation. Nature. (2019) 574:575–80. doi: 10.1038/s41586-019-1678-1, PMID: 31645732 PMC6818755

[48] Nelson VL Nguyen HCB Garcìa-Cañaveras JC Briggs ER Ho WY DiSpirito JR . PPARγ is a nexus controlling alternative activation of macrophages via glutamine metabolism. Genes Dev. (2018) 32:1035–44. doi: 10.1101/gad.312355.118, PMID: 30006480 PMC6075146

[49] Ganta VC Choi MH Kutateladze A Fox TE Farber CR Annex BH . A microRNA93-interferon regulatory factor-9-immunoresponsive gene-1-itaconic acid pathway modulates M2-like macrophage polarization to revascularize ischemic muscle. Circulation. (2017) 135:2403–25. doi: 10.1161/CIRCULATIONAHA.116.025490, PMID: 28356443 PMC5503157

[50] Liao ST Han C Xu DQ Fu XW Wang JS Kong LY . 4-Octyl itaconate inhibits aerobic glycolysis by targeting GAPDH to exert anti-inflammatory effects. Nat Commun. (2019) 10:5091. doi: 10.1038/s41467-019-13078-5, PMID: 31704924 PMC6841710

[51] Shi X Zhou H Wei J Mo W Li Q Lv X . The signaling pathways and therapeutic potential of itaconate to alleviate inflammation and oxidative stress in inflammatory diseases. Redox Biol. (2022) 58:102553. doi: 10.1016/j.redox.2022.102553, PMID: 36459716 PMC9713374

[52] Wu B Zhao TV Jin K Hu Z Abdel MP Warrington KJ . Mitochondrial aspartate regulates TNF biogenesis and autoimmune tissue inflammation. Nat Immunol. (2021) 22:1551–62. doi: 10.1038/s41590-021-01065-2, PMID: 34811544 PMC8756813

[53] Luo Y Qi X Zhang Z Zhang J Li B Shu T . Inactivation of Malic enzyme 1 in endothelial cells alleviates pulmonary hypertension. Circulation. (2024) 149:1354–71. doi: 10.1161/CIRCULATIONAHA.123.067579, PMID: 38314588

[54] Wang R Dillon CP Shi LZ Milasta S Carter R Finkelstein D . The transcription factor Myc controls metabolic reprogramming upon T lymphocyte activation. Immunity. (2011) 35:871–82. doi: 10.1016/j.immuni.2011.09.021, PMID: 22195744 PMC3248798

[55] Dang CV Le A Gao P . MYC-induced cancer cell energy metabolism and therapeutic opportunities. Clin Cancer Res. (2009) 15:6479–83. doi: 10.1158/1078-0432.CCR-09-0889, PMID: 19861459 PMC2783410

[56] Lee CM He CH Nour AM Zhou Y Ma B Park JW . IL-13Rα2 uses TMEM219 in chitinase 3-like-1-induced signalling and effector responses. Nat Commun. (2016) 7:12752. doi: 10.1038/ncomms12752, PMID: 27629921 PMC5027616

[57] Chen Y Zhang S Wang Q Zhang X . Tumor-recruited M2 macrophages promote gastric and breast cancer metastasis via M2 macrophage-secreted CHI3L1 protein. J Hematol Oncol. (2017) 10:36. doi: 10.1186/s13045-017-0408-0, PMID: 28143526 PMC5286803

[58] Wang J Zhou Y Hu J Han J Feng J Guo K . Characteristic genes and immune landscape of interstitial cystitis. PloS One. (2025) 20:e0320249. doi: 10.1371/journal.pone.0320249, PMID: 40435311 PMC12119006

[59] Wu L Wang T Bai M Chen J Ning J Jin Q . Causal role of common autoimmune diseases in interstitial cystitis/bladder pain syndrome: Mendelian randomization (MR) study. Medicine. (2025) 104:e41484. doi: 10.1097/MD.0000000000041484, PMID: 39993091 PMC11857018

[60] Liu J Zhang Y Li S Sun F Wang G Wei D . Bioinformatics analysis of the Hub genes and key pathways of interstitial cystitis pathogenesis. Neurourology urodynamics. (2020) 39:133–43. doi: 10.1002/nau.24196, PMID: 31663162

[61] Inal-Gultekin G Gormez Z Mangir N . Defining molecular treatment targets for bladder pain syndrome/interstitial cystitis: uncovering adhesion molecules. Front Pharmacol. (2022) 13:780855. doi: 10.3389/fphar.2022.780855, PMID: 35401223 PMC8990855

[62] Mwale PF Hsieh CT Yen TL Jan JS Taliyan R Yang CH . Chitinase-3-like-1: a multifaceted player in neuroinflammation and degenerative pathologies with therapeutic implications. Mol neurodegeneration. (2025) 20:7. doi: 10.1186/s13024-025-00801-8, PMID: 39827337 PMC11742494

