# Supplementary material for: Enhanced CHI3L1 promotes macrophage activation in persistent inflammatory events of ulcerative interstitial cystitis
Source: Front Immunol. 2026 Jan 29;17:1716297. doi: 10.3389/fimmu.2026.1716297 (PMC12894025; doi:10.3389/fimmu.2026.1716297)
Supplement: Supplementary file 1 [file Table1.docx]

**Supplement table 1: Primers used for qPCR**

| **Gene** | **Species** | **Primer sequence (5′-3′)** |
| --- | --- | --- |
| β-actin | Mouse | Forward: GGCTGTATTCCCCTCCATCG  Reverse: CCAGTTGGTAACAATGCCATGT |
| Il6 | Mouse | Forward: TCCATCCAGTTGCCTTCTT  Reverse: ACGATTTCCCAGAGAACATG |
| Tnf | Mouse | Forward: CGTCAGCCGATTTGCTATCT  Reverse: CGGACTCCGCAAAGTCTAAG |
| GAPDH | Mouse | Forward: GTCAAGGCCGAGAATGGGAA  Reverse: CTCGTGGTTCACACCCATCA |
| Chi3l1 | Mouse | Forward: ATGACATCGCCCAGATAGCC  Reverse: GGGATGCCCATCAGTAGCTT |
