# Supplementary material for: Enhanced CHI3L1 promotes macrophage activation in persistent inflammatory events of ulcerative interstitial cystitis
Source: Front Immunol. 2026 Jan 29;17:1716297. doi: 10.3389/fimmu.2026.1716297 (PMC12894025; doi:10.3389/fimmu.2026.1716297)
Supplement: Supplementary file 2 [file Table2.docx]

| **Group** | **Number** | **Vector plasmid information** |
| --- | --- | --- |
| OE | TSC666-1 | pPB[Exp]-EF1A-EGFP>CAG-mChi3l1[NM_007695.4]>PGK-Puro |
| NC | PBC03 | pPB[Exp]-EF1A-EGFP>CAG-[ORF-stuffer]>PGK-Puro |
